# Magnet dislocation during 3 T magnetic resonance imaging in a pediatric case with cochlear implant^☆^

**DOI:** 10.1016/j.bjorl.2016.04.025

**Published:** 2016-06-18

**Authors:** Abdulkadir Özgür, Engin Dursun, Fatma Beyazal Çeliker, Suat Terzi

**Affiliations:** a Recep Tayyip Erdogan University, Medical Faculty, Department of Otorhinolaryngology, Rize, Turkey; b Recep Tayyip Erdogan University, Medical Faculty, Department of Radiology, Rize, Turkey

## Introduction

Cochlear implant (CI) is a surgically implanted device designed for treatment of severe sensorineural hearing loss in pediatric and adult patients. With the advanced technology and satisfying results, the indications of CI are gradually expanding.^1^ But the increase in implantation has brought with it some problems. One of the major problems faced by CI users is those experienced during magnetic resonance imaging (MRI). MRI is a standard radiological imaging method used for diagnosis of many diseases. The magnetic field generated during imaging can lead to unwanted problems such as device failures, unwanted electrical currents, displacement of the device and demagnetization.^2^, ^3^ A magnet dislocation case seen after 3 T MRI is presented with the review of literature in the present report.

## Case report

A four-year-old male patient with cochlear implant was referred to our clinic with the complaints of pain having occurred during MRI and failure to be able to replace the external part of cochlear implant over the internal part. The 3 T MRI had been performed the day before. According to information received from his parents, the patient had bilateral profound congenital hearing loss. He had undergone tumor resection from his right ear 15 months earlier. Total hearing loss had occurred in his right ear after tumor surgery. He had been diagnosed with Langerhans cell histiocytosis. Six months after the tumor surgery, a cochlear implant (Nucleus Freedom Straight CI24RE) was implanted in his left ear for sensorineural hearing loss. During his follow-up, complaints of excessive fluid intake and frequent urination emerged. Further evaluation confirmed a diagnosis of diabetes insipidus and an MRI was planned with suspicion of intracranial spread of Langerhans cell histiocytosis. A head bandage was applied before the MRI that took place in another center but the patient experienced pain during the MRI procedure; the imaging process was terminated immediately. As the external piece of device could not be replaced, the patient was referred to our clinic.

**Figure 1:**
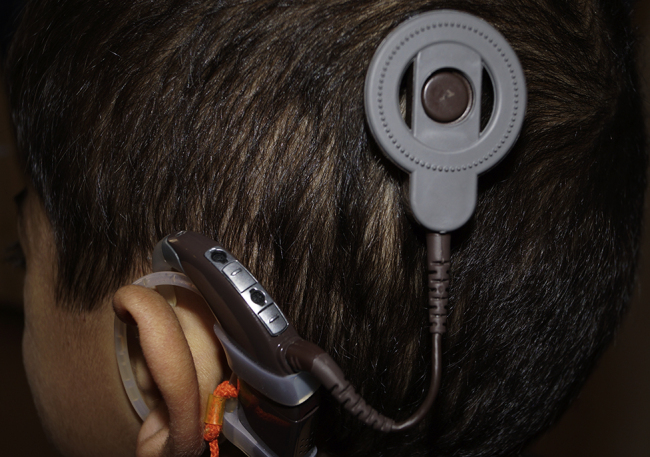
After the reversal of the external magnet, the external part appears to hold on to the internal part.

**Figure 2:**
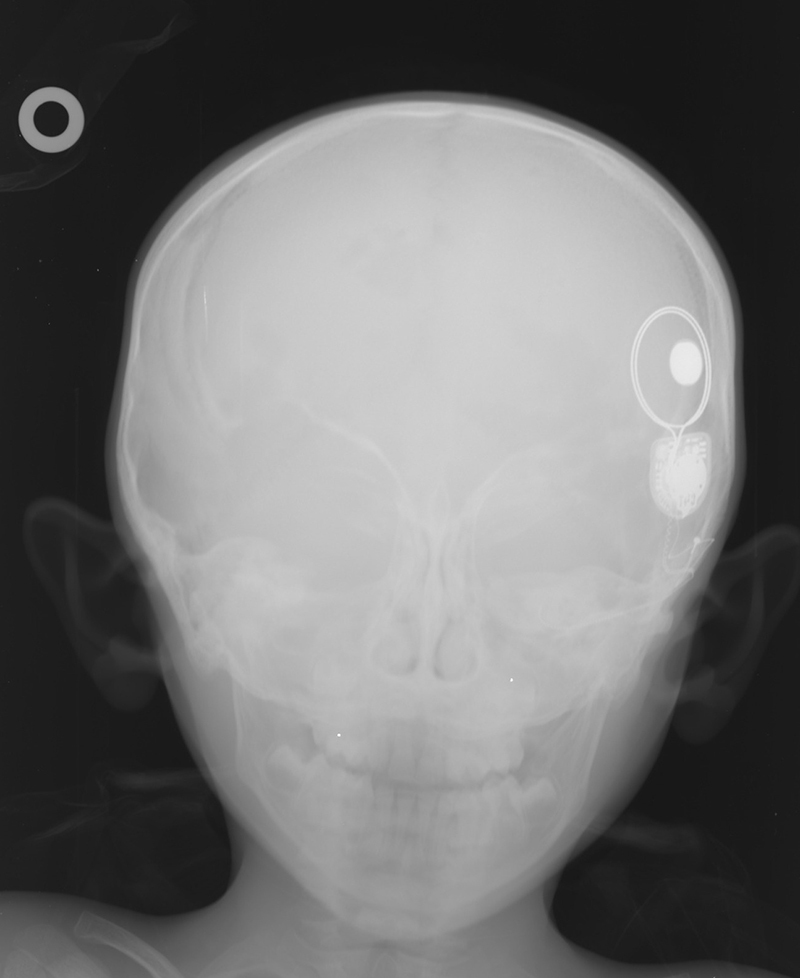
Preoperative radiography of the patient.

The physical examination showed a swelling in the area where the magnet was estimated to be located. The magnet had turned upside down, the external part was reversed (inside facing out) and still attracting to the internal part (Fig. 1). After reversing the external magnet, it was attached to the internal part. In this case, it was found that the stimulation had been restored. The patient was examined radiographically and a minimal shift was observed in the position of the magnet (Fig. 2). Considering the status of patient's disease, a new MRI was planned after removing the magnet.

**Figure 3:**
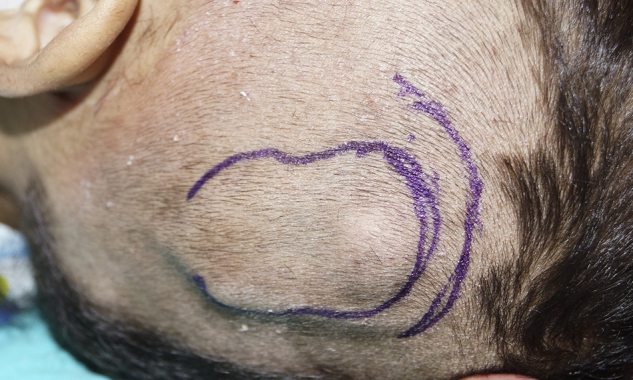
Marking the boundaries of the inner part for planning the skin incision.

**Figure 4:**
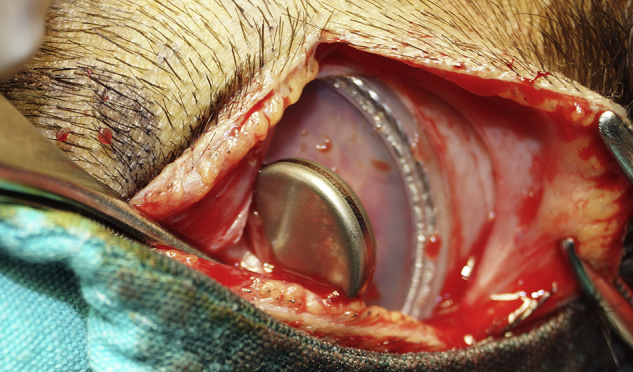
View of the internal part and reversed internal magnet.

**Figure 5:**
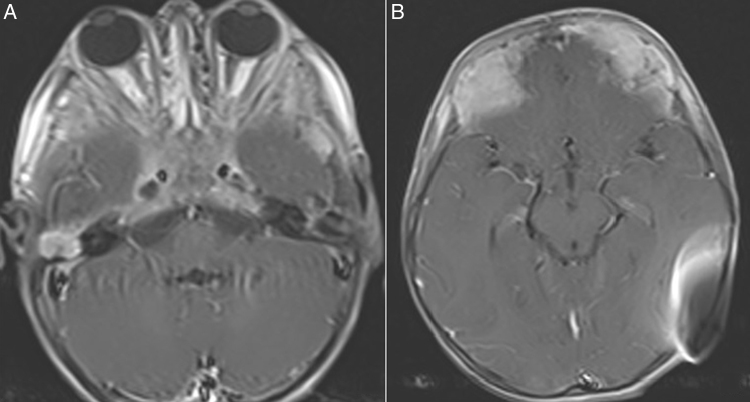
Lesions showing contrast enhancement in the right mastoid bone, skull base and anterior fossa with artifacts created by remainder of the internal part after intravenous contrast medium administration.

A skin incision was performed under sedoanalgesia as it would not pass through the skin contact area of the device's external and internal part (Fig. 3). After incising the skin, subcutaneous tissue and periosteal layer, the internal part of magnet was reached (Fig. 4). The magnet was turned upside down and was found set apart from its bed superiorly. The magnet was removed and the incision was closed. To avoid hematoma, a compression bandage was applied and then an MRI was performed at 1.5 T. The MRI showed that the tumor had infiltrated the bilateral petrous apex, clivus, cavernous sinus, and the anterior fossa. Additionally, it had obliterated the frontal sinuses and had extended to fronto-parietal calvarial region. The tumor showed diffuse heterogeneous contrast enhancement after intravenous contrast medium administration. Isointense areas in T1 imaging and heterogeneous hypointense areas in T2 imaging were observed (Fig. 5). The MRI procedure was completed without any problems. The patient was recommended a new magnet placement in order to use the implant again, but the parents refused a surgical intervention since the patient was in a bad general condition. They stated that they would decide the intervention after the completion of treatment. The patient currently remains under chemotherapy for treatment of the tumor.

## Discussion

The risk of a complication is very low for cochlear implants. Vestibular complaints (3.9%), device failure (3.4%), loss of taste (2.8%) and skin problems (1.3%) are the most common documented long-term complications in the meta-analysis studies.^4^ Generally, solutions to these problems have been approached through modifications in the surgical technique.^5^ One of the problems more often seen, and one which cannot be solved by surgical modification, is that experienced in MRI procedures. An MRI is an imaging technique that is widely used for the diagnosis of many diseases such as stroke, neurodegenerative diseases and tumors. The magnetic field occurring during the MRI can lead to problems such as displacement of the device and demagnetization.^1^, ^2^, ^3^ Since an MRI at 3 T or above yields a higher-quality image, the use of these devices has become widespread. However, as the strength of the resulting magnetic field increases, the problems faced by CI users increases as well.^6^

In a study published in 2014, Hassepass et al. reported that they had performed 22 magnet revision surgeries out of 2027 cochlear implant patients. Twelve (52.2%) of these patients had a dislocation that had occurred after MRI.^7^ Additionally, similar magnet dislocation cases after MRI have been reported.^1^, ^8^, ^9^ The images were obtained at 1.5 T MR device in these presented cases. The magnet dislocation in our case was observed at 3 T MRI, which had a stronger magnetic field than 1.5 T MRI. The magnet was turned upside-down and changed its polarization similar to other cases in the literature. A tight bandage application is considered to be sufficient during an MRI at 1.5 T, especially for the new generation cochlear implant systems. However, removing the magnet is advised for imaging above 1.5 T. The manufacturer of the cochlear implant system used by our patient had recommended the removal of the magnet for MRI at 3 T. However, only a tight headband had been used during MRI and the procedure was terminated due to pain.

The problem faced with the change of a magnet's polarization was solved by changing the direction of external magnet in a case presented by Jeon et al.^1^ Titanium plates were inserted after removing the magnet in another two cases.^8^, ^9^ In our case, for the detection of spread of tumor and to reduce artifact, the MRI was performed after the magnet had been removed. Skin problems are the most important problems encountered in magnet revision surgery. The incision should not pass over the internal part in order to reduce skin problems.^10^ In the present case, the skin incision was performed so that it would not pass through the skin contact area of the device's external and internal parts in order to reduce wound complications. The postoperative period was uneventful in terms of wound problems.

## Conclusion

One of the issues related to cochlear implant users is complications that may occur during MRI, which is widely used as a standard imaging method nowadays. To the best of our knowledge, this is the first case of magnet dislocation seen after 3 T MRI. For prevention of such complications during MRI, patients and their relatives should be informed in detail about the possible risks when using higher resolution MRI.

## Conflicts of interest

The authors declare no conflicts of interest.
